# Chronic Obstructive Pulmonary Disease: Effects beyond the Lungs

**DOI:** 10.1371/journal.pmed.1000220

**Published:** 2010-03-16

**Authors:** Peter J. Barnes

**Affiliations:** National Heart & Lung Institute, Imperial College, London, United Kingdom

## Abstract

Peter Barnes discusses the growing epidemic of chronic obstructive pulmonary disease (COPD), especially in developing countries and among nonsmokers.

Chronic obstructive pulmonary disease (COPD) is a growing global epidemic that is particularly important in developing countries. Morbidity and mortality from COPD will rise as populations age and mortality from cardiovascular and infectious diseases falls. Whereas cigarette smoking is the commonest cause of COPD in developed countries, COPD is also seen in nonsmokers, particularly in developing countries, yet very little is know about this form of COPD. COPD is primarily characterized by the presence of airflow limitation resulting from inflammation and remodelling of small airways and is often associated with lung parenchymal destruction or emphysema. It is increasingly recognised that COPD extends beyond the lung and that many patients have several systemic manifestations that can further impair functional capacity and health-related quality of life [1]. In addition, COPD is associated with several other diseases, such as cardiovascular diseases, osteoporosis, diabetes, and metabolic syndrome, more commonly than expected by chance. These associations are greater than expected from common aetiological factors, such as smoking, suggesting that these comorbidities may be causally associated with the mechanisms of COPD. Systemic effects and comorbidities of COPD increase both the risks of hospitalisation and mortality and the costs, and are therefore a topic of increasing concern [2]. Indeed, cardiovascular disease and lung cancer are the commonest causes of death in patients with COPD [2],[3]. Although comorbidities are more commonly seen in association with severe COPD, they may also be associated with milder or earlier disease.

## Mechanisms: The Role of Systemic Inflammation

**Figure 1:**
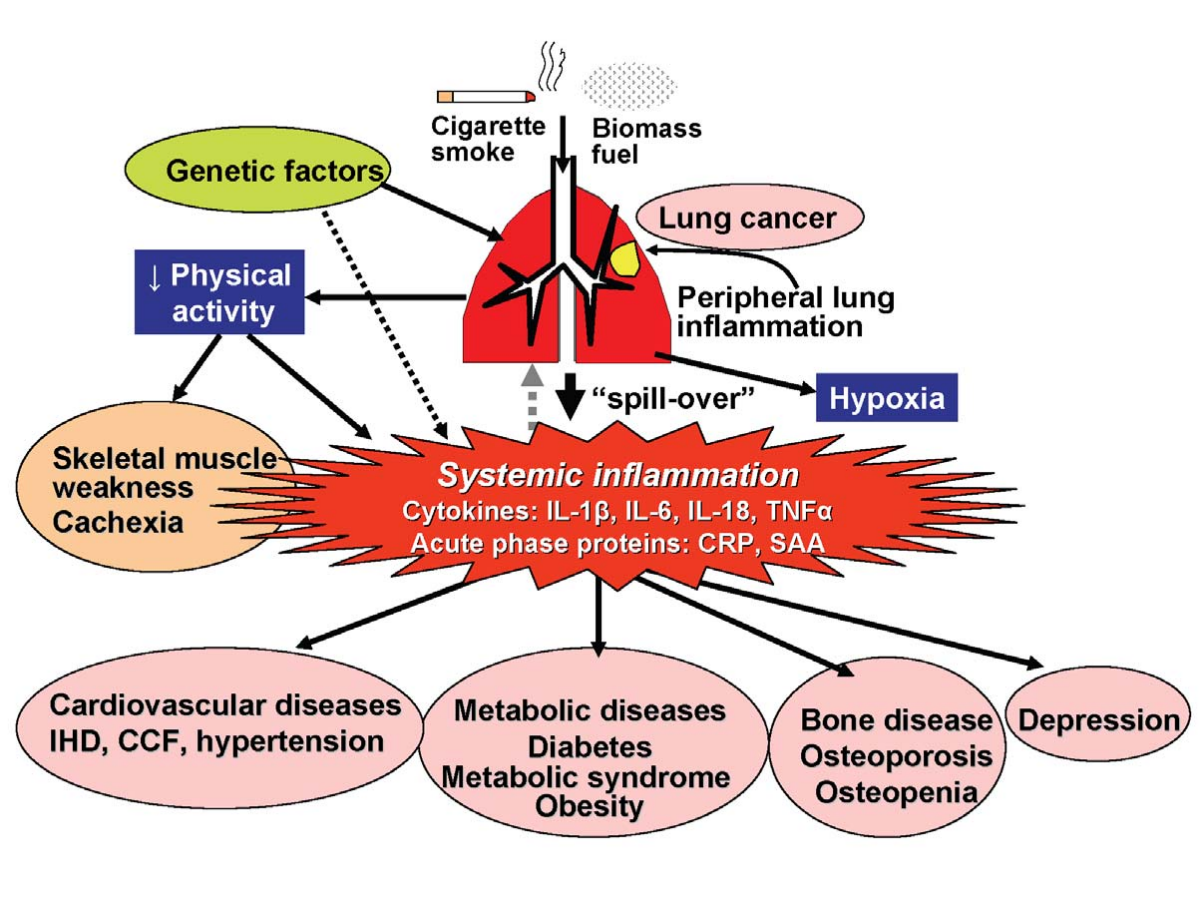
Patients with COPD have peripheral lung inflammation that may spill over into the systemic circulation, leading to skeletal muscle weakness and cachexia and increasing propensity to cardiovascular, metabolic, and bone diseases, and depression. There is an increase in circulating cytokines, including IL-1β, IL-6, IL-18, and TNFα, as well as acute-phase proteins, such as CRP and serum amyloid A. Peripheral lung inflammation may also increase the risk of developing lung cancer. There may be genetic predisposition to developing COPD in smokers, which may be shared with genetic susceptibility to comorbid diseases. Airway obstruction reduces physical activity and causes hypoxia, which may contribute to skeletal muscle weakness as well as to comorbid diseases.

The mechanism linking COPD to systemic manifestations and comorbidities is not yet certain, but a potential mechanism is systemic inflammation. Other possible mechanisms (which are not mutually exclusive) include shared genetic predispositions, physical inactivity secondary to airway obstruction, and chronic hypoxia. Several inflammatory cytokines, including tumour necrosis factor-α (TNFα), interleukin(IL)-6, CXCL8 (IL-8), and IL-18, and acute phase proteins, such as C-reactive protein (CRP), serum amyloid A, and fibrinogen, are increased within the circulation of patients with COPD, particularly during exacerbations [4]. The cytokines that are increased in the circulation are also increased in sputum and bronchoalveolar lavage fluid of COPD patients, suggesting that the systemic cytokines represent an overspill of inflammatory mediators from the peripheral lung (Figure 1) [1]. Since the circulating cytokines found in COPD are common to many inflammatory diseases, this mechanism is difficult to verify. Recently, however, surfactant protein D, which is produced exclusively by type II pneumocytes, were found to be elevated in the circulation of COPD patients, providing more direct evidence for the overspill hypothesis [5].

However, there is not a close correlation between the concentration of mediators in sputum and blood, suggesting that other factors are also involved. Physical inactivity as a consequence of progressive airflow limitation may be an important factor in promoting some comorbidities, such as skeletal muscle weakness, osteoporosis, and cardiovascular disease. There is also increasing evidence that emphysema represents accelerated ageing of the lung and that many of the comorbidities associated with COPD, such as osteoporosis, cardiac failure, and diabetes, are also diseases of accelerated ageing and may be a consequence of reduced synthesis of anti-ageing molecules, such as sirtuins [6]. There may be genetic factors that increase susceptibilities to develop certain comorbidities and there may be common genetic factors linked to increased susceptibilities of smokers to develop COPD. Another view is that several diseases may contribute to systemic inflammation and that this affects the lung, resulting in worsening of the structural changes of COPD [7]. However, COPD can certainly occur without any evidence of systemic inflammation, so this hypothesis seems less likely.

## COPD and Cardiovascular Disease

Cardiovascular disease is a major cause of mortality and morbidity in patients with COPD. Although there are common causal factors including smoking and sedentarism, the increase in cardiovascular disease is independent of these known risk factors [2]. Recently, several studies have shown that COPD patients have increased arterial stiffness, which may explain the epidemiological link between reduced FEV_1_ (forced expiratory volume in 1 second) and cardiovascular mortality [8]. The mechanism for increased arterial stiffness is not yet certain but could reflect systemic endothelial cell dysfunction as a result of systemic inflammation. However, a recent study was not able to demonstrate any defect in endothelial function in COPD patients with arterial stiffness, suggesting that it may be due to an abnormality in the arterial wall [9]. Systemic inflammation may predispose to atherosclerotic plaques, which may account for the high prevalence of myocardial infarction in patients with COPD.

Chronic heart failure is also common amongst COPD patients and in a recent study over 20% of patients were found to have undiagnosed left ventricular failure by magnetic resonance imaging [10]. It may be difficult to diagnose heart failure in COPD patients because of the overlap in symptoms, but measurement of plasma B-type natriuretic peptide concentrations is useful in detecting cardiac failure.

## COPD and Metabolic Diseases

There is an increased risk of diabetes in COPD patients [2]. Systemic inflammation, and particularly the proinflammatory cytokines TNF-α and IL-6, may induce insulin resistance. Metabolic syndrome, characterised by central obesity, diabetes, hypertension, and hyperlipidaemia, is also known to occur with COPD. In a recent study of COPD patients, metabolic syndrome was found in almost half of the patients irrespective of disease stage and was associated with increased markers of systemic inflammation, including IL-6, CRP, and fibrinogen [11]. In an epidemiological study in China metabolic syndrome was identified in approximately 20% of patients with COPD, and this correlated most closely with central obesity [12]. Cachexia and weight loss has also been associated with COPD, particularly in severe disease and may be related to increased concentrations of certain cytokines, such as TNF-α. There is selective loss of skeletal muscle, measured as fat-free mass, and this is associated with a selective loss of type IIA fibres. To some extent this muscle wasting is explained by physical inactivity and tends to be more pronounced in the lower limbs, but there may also be metabolic mechanisms such as systemic oxidative stress and circulating inflammatory cytokines that accelerate this loss.

## COPD and Bone Disease

Several studies have shown a very high prevalence of osteoporosis and low bone mineral density in patients with COPD, even with milder stages of disease [13]. In a cross-sectional study the prevalence of osteoporosis was 75% in patients with GOLD (Global Initiative on Obstructive Lung Disease) stage IV disease and was strongly correlated with reduced fat-free mass, especially in women. Vertebral compression fractures are relatively common amongst COPD patients, and the resultant increased kyphosis may further reduce pulmonary function. There are shared risk factors between COPD and osteoporosis, including cigarette smoking and physical inactivity and high doses of inhaled corticosteroids, but COPD itself appears to be a risk factor for osteoporosis and osteopenia. Osteoporosis is also linked to arterial stiffness as well as COPD and this has suggested a common aetiology as a result of systemic inflammation [8]. Inflammatory cytokines, such as IL-1β, IL-6, and TNF-α, inhibit osteoclasts and may promote bone resorption.

## COPD and Psychiatric Diseases

Anxiety and depression are remarkably common in COPD patients due in part to their social isolation, but appear to be more common than in other chronic disabling diseases. Major depression that requires medical intervention occurs in 20%–40% of COPD patients, but there are wide variations in the incidence of depression, as different assessment tools have been used, and the diagnosis may be overlooked, as there is an overlap of symptoms. Depression worsens health status and increases mortality in COPD patients and it should therefore be treated appropriately. The mechanisms of depression are multifactorial, but there is growing evidence that systemic inflammation may result in depression and IL-6 appears to play a particularly important role in humans and in animal models of depression.

Cognitive dysfunction is also increasingly recognised in patients with COPD, especially in severe disease, and this may have an important impact on self management and adherence to therapy [14].

## COPD and Lung Cancer

Patients with COPD are 3 to 4 times more likely to develop lung cancer than are smokers with normal lung function. Lung cancer is found in 40%–70% of patients with COPD, particularly in severe disease, and is a common cause of death in COPD patients [15]. Lung cancer is also more common in patients with COPD who have never smoked in a large prospective trial of almost half a million nonsmokers [16]. The most likely explanation for the increased risk of lung cancer in COPD is the presence of chronic inflammation, with increased production of growth factors and angiogenic factors. Stopping smoking in COPD patients reduces but does not eliminate the risk of lung cancer, probably because inflammation persists even after smoking cessation [17].

## Implications for Management

The recognition that COPD is a multidimensional disease with numerous comorbidities and systemic effects has several important consequences for long-term management of this disease. Although there are national and international guidelines for the management of COPD, they deal mainly with the pulmonary aspects of the disease and do not address the management of comorbid diseases, which are managed by other sets of guidelines drawn up by different groups of specialists. There needs to be more integration of these guidelines, indicating how comorbidities are best managed in COPD patients and what the best choice of therapy should be, taking into account drug interactions and propensity to adverse effects as a result of the comorbid disease. In turn, other specialists should be aware that COPD is commonly comorbid with cardiovascular and metabolic diseases, and this may only become apparent if spirometry is performed, as COPD is very often undiagnosed in these patients. In other words, a more integrated approach to management is required.

It is now emerging that several treatments used in comorbid diseases may have beneficial effects in COPD. Observational studies suggest that COPD patients treated with statins, angiotensin-converting enzymes, and beta-blockers may have improved survival and reduced hospitalisation from exacerbations [1]. However, because of selection biases inherent in these studies, it is important to conduct placebo-controlled randomised clinical trials to determine where these treatments may improve COPD outcomes. A large trial of statin therapy in COPD patients is currently underway to address this issue (ClinicalTrials.gov NCT01061671). Peroxisome proliferator activator receptor (PPAR)-γ agonists, developed to treat diabetes, may also have therapeutic potential in COPD as anti-inflammatory and anti-fibrotic treatments.

Treatments currently used for management of COPD might have effects on systemic manifestations and comorbidities, but this has not often been clearly assessed. Smoking cessation and reduction of exposure to biomass fuel smoke are important for their beneficial effects on comorbidities as well as pulmonary disease. In a large trial of the anticholinergic bronchodilator tiotropium bromide in COPD patients (UPLIFT) there was a significant reduction in cardiovascular mortality [18]. By contrast, an inhaled corticosteroid had no effect on cardiovascular mortality [3]. Several new treatments for COPD are now in development and, in addition to any effect on the pulmonary disease, it will be important to assess whether these treatments affect systemic manifestations and comorbid diseases.

## Conclusions and Future Directions

COPD is a multidimensional disease, with several systemic manifestations and associations with a number of comorbid diseases. The most likely link between COPD and these extrapulmonary conditions is spillover of inflammatory mediators from the lung, as systemic inflammation is associated with skeletal muscle wasting and cachexia as well as with cardiovascular, metabolic, and bone diseases. More research is needed to understand the links between these diseases and to search for common treatable components. It seems likely that treatments, such as statins, that are already used to manage cardiovascular and metabolic diseases might also provide benefit in COPD patients, although it is important that randomised placebo-controlled trials be conducted to confirm this possibility. It is important to consider how the existence of a comorbid disease may affect the management of the patient who also has COPD. Guidelines are needed that recognise the association of these various diseases and provide advice on management, as well as defining questions for future clinical research. In turn, it is important for specialists in nonpulmonary areas to recognise that COPD may commonly occur in association with certain diseases in their speciality and to make the diagnosis using spirometry so that appropriate treatment may be instituted. Treatments already used in the treatment of COPD might also be beneficial in some comorbid diseases. New therapies should also be considered as potentially beneficial to systemic manifestations and comorbidities. For example, an effective inhaled anti-inflammatory therapy may improve comorbidities by reducing the overspill of inflammatory mediators from the lung that contribute to systemic inflammation. Alternatively, an oral anti-inflammatory treatment, as well as suppressing inflammation on the respiratory tract, may directly reduce systemic inflammation. It is clear that much more clinical and basic research is needed to understand the complexity of COPD so that more effective management of COPD and its various comorbidities is possible in the future.

## Abbreviations

COPD: chronic obstructive pulmonary disease
CRP: C-reactive protein
FEV_1_: forced expiratory volume in 1 second
IL: interleukin
TNF: tumour necrosis factor
